# Population genetics and GWAS: A primer

**DOI:** 10.1371/journal.pbio.2005485

**Published:** 2018-03-16

**Authors:** Greg Gibson

**Affiliations:** School of Biological Sciences, Georgia Institute of Technology, Atlanta, Georgia, United States of America

## Abstract

This primer provides some background to help non-specialists understand a new theoretical evolutionary genetics study that helps explain why thousands of variants of small effect contribute to complex traits.

If population genetics is the study of allele frequencies and quantitative genetics the study of allelic effects, then evolutionary genetics aims to understand how they interact over time. Matching of models to data has traditionally been constrained by the bias of observation toward common alleles that have large effects, limiting our ability to address questions such as “what maintains variation in natural populations,” “how many genes influence a trait,” or “to what extent do drift and selection influence allele frequencies” [1]. Now that we are well into the deep sequencing and genome-wide association study (GWAS) era, that situation is changing and it is now feasible to test models against comprehensive empirical data. The paper by Simons et al in this issue [2] goes a long way toward building a framework for synthesizing population genetics and the new quantitative genetics.

Two findings differentiate GWAS from much of 20th century genetics: the extraordinarily high polygenicity of traits and the dearth of evidence for interaction effects, whether among genes or with the environment [3]. Whereas a typical genetic mapping experiment in a cross between two lines or in a pedigree reveals perhaps 10–20 so-called quantitative trait loci (QTL), each explaining perhaps 5% of the phenotypic variation, GWAS on tens or hundreds of thousands of unrelated individuals discover hundreds and implicate many thousands of loci, each explaining a fraction of a percent of the variance. From height to educational attainment, diabetes to schizophrenia, human geneticists now embrace the infinitesimal model with little exception, accepting that as many as 5% of all common variants in an even greater percentage of genes might associate with any given trait [4], along with an unknown number of rare variants of larger effect [5].

Despite this complexity, there are also signs that what is called the “genetic architecture” does differ among traits, namely that different numbers of genes with different spectra of allele frequencies (and perhaps propensity to interact) associate with each trait [6]. This is partly reflected in the fact that the heritability, or proportion of the variance in a population that is attributable to genetic differences, varies, but is also true of traits that have similar heritabilities. Why?

Very soon after the first GWAS studies appeared 10 years ago, it was recognized that considerably less genetic variation was being discovered than expected, given well-validated heritability estimates [7]. This led to much spilled ink on the missing heritability problem, but once it is recognized that most genetic effects are very small, it becomes apparent that GWAS is generally underpowered and that it is more a hidden heritability problem [8]. If your keys drop out of your purse in the subway, they are likely to be forever missing, but if they are lost under the pile of papers on your desk, they are temporarily hidden. Concerted effort will find them. In GWAS, this means larger sample sizes or more subtle study designs, and indeed ever-larger meta-analyses do seem to discover more and more variants. Whether or not this will continue to be the case with studies of millions of people is an important question [6], so it is notable that one of the implications of Simons et al [2] is that there may be a limit to the discovery of very small-effect loci, which will remain inferred but forever missing.

Contrasting these interpretations requires accurate estimation of the distribution of allelic effects. Three general approaches have been used to do so, broadly extrapolation, interpolation, and simulation. Extrapolation studies [9] take the observed distribution of GWAS hits for a given sample size and estimate how many more loci would be uncovered if the sample size is increased—they make no assumptions about evolutionary parameters. Interpolation is based on modeling the relationship between genetic similarity and phenotypic similarity without attempting to identify individual loci [10,11], but it turns out that how much so-called SNP-based heritability (the total amount of variation attributable to common variants) is inferred depends on your assumptions about, among other things, the relationship between allele frequency and effect size. Simulation attempts to evolve populations in silico in the presence of mutation, selection, and diverse demographic scenarios, matching the genetics of hypothetical traits to observed architectures. One prominent example [12] concluded that GWAS findings are consistent with a broad but nevertheless constrained range of parameters, concluding that only weak coupling between selection and phenotypic effects was required to explain observed allele frequency distributions for disease.

The reason why allele frequencies matter is that the amount of variance a biallelic polymorphism contributes is equal to 2*pqa*^2^, where *p* and *q* are the two allele frequencies, and *a* is the effect size of the allele in standard deviation units (sdu’s). Thus, if an SNP with a frequency of 0.5 adds an average of 7 mm to a person’s height, which is 0.1 sdu, then it accounts for about 0.5% of the variation in the population, which it turns out would be a large contribution. One with a frequency of 0.1 adding 1 mm would, by contrast, explain 0.0037% of the variation, which is more typical of observed contributions. As shown by the blue curve in Fig 1A, the rarer the allele, the less variance explained (and the less likely it is to be discovered), although several lines of evidence now suggest that effect sizes tend to be larger for less common alleles. These include empirical distributions of effect sizes [13], interpolation approaches that capture more heritability under this scenario [14], and the observation that very rare variants are underrepresented in the human genome [15].

**Fig 1:**
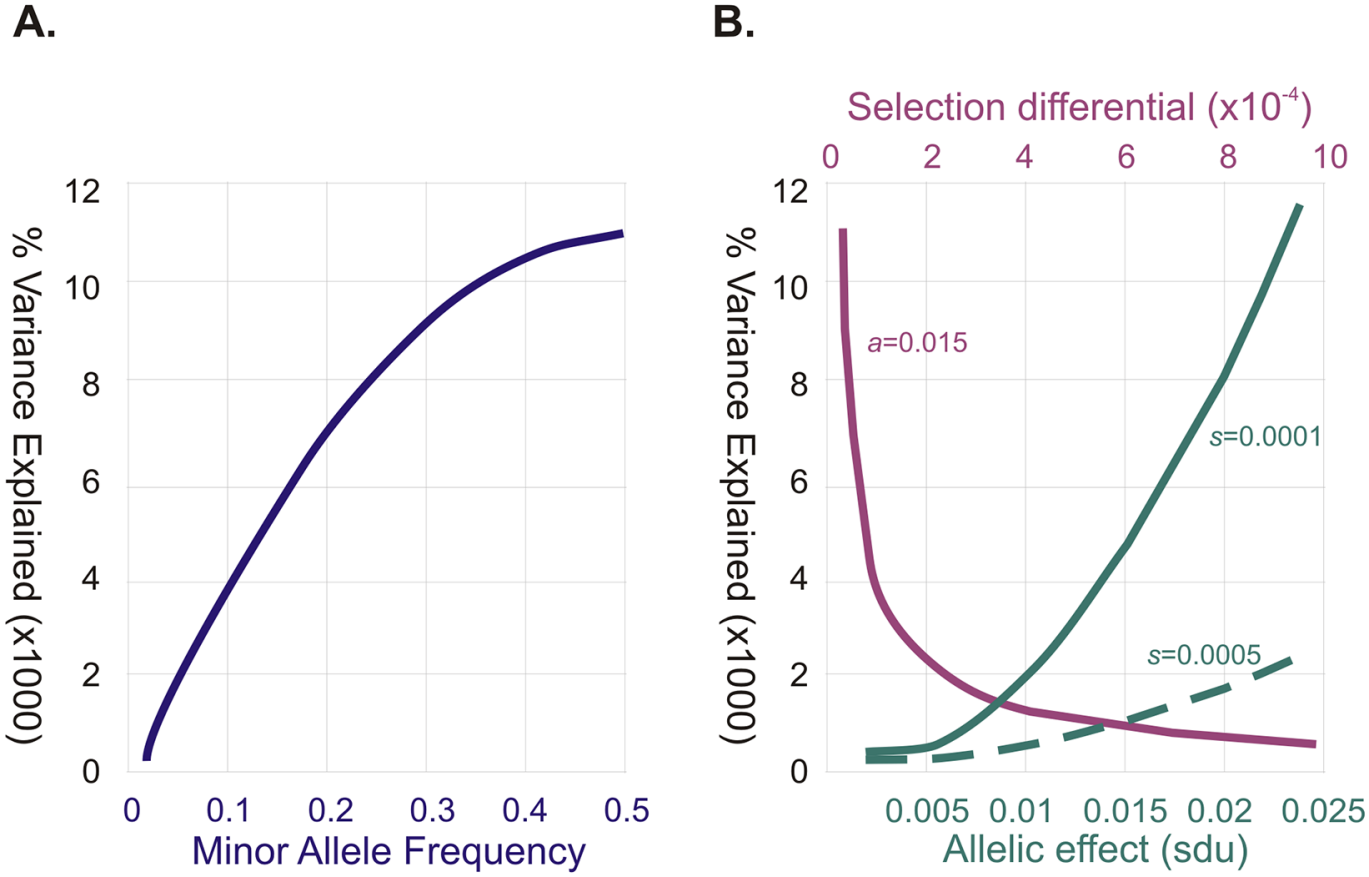
Relationships between allele frequency, selection differential, effect size, and variance explained. (A) The blue curve shows how the percent of variance explained varies as a function of minor allele frequency, *p*, namely %Variance Explained = 2*p*(1-*p*)*a*^2^. The curve assumes 1,000 alleles, each with an additive contribution, *a*, of 0.015 sdu’s (about 1 mm of human height). (B) Simons et al [2] demonstrate that for relatively strongly selected alleles, the variance explained per site, *v*_s_, is a function of the contribution to fitness in a population size *N* with degree of pleiotropy *n*. Specifically, *v*_s_ = 2*w*^2^/*nN*, and because the selection coefficient s = *a*^2^/w^2^, then *v*_s_ = 2*a*^2^/s*nN*. The magenta curve approximates the expected selection coefficient consistent with *a* = 0.015 in a population with an effective size of 10,000 alleles affecting 10 traits, and *v*_s_ expected for 1,000 alleles to produce the indicated %Variance Explained: as selection increases, less variance is explained because the allele frequencies drop. Alternatively, the solid green curve assumes a constant *s* = 10^−4^ and shows the effect sizes consistent with variation explained, while the dashed green curve shows how increasing the selection pressure 5-fold reduces the amount of variance that can be maintained. Alleles explaining on average 0.01% of the variance under these scenarios could be consistent with substitution effects of 0.015 sdu, intermediate selection coefficients approximately 5×10^−5^ leading to minor allele frequencies about 0.33; or with *a* = 0.023, and *s* = 10^−4^ and *p* ~ 0.1; with *a* = 0.05, and *s* = 5 × 10^−4^, p ~ 0.02, and so forth. sdu, standard deviation unit.

The latter finding is parsimoniously attributed to purifying selection: larger effect alleles are more likely to be deleterious and less likely to rise in frequency in the gene pool. It is easy to think about purifying selection as selection against deleterious variants that promote disease, but it turns out that for a substantial proportion of GWAS hits, the “risk” allele is either the more common one and/or the ancestral one. Both properties belie the simple interpretation that selection against disease is the major factor shaping the genetic architecture of traits. Rather, many consider stabilizing selection to be more prominent [16], namely selection against variants that tend to perturb the phenotype away from an optimal intermediate value, either in the high or low direction. This is certainly true of transcript abundance [17], and because the majority of GWAS variants are regulatory, affecting gene expression, it follows that stabilizing selection is pervasive. An additional twist is that we also think that pleiotropy is the norm, namely that most functional variants impact multiple traits [18]. These may be independent, perhaps height and intelligence due to gene activity in bone and brain, or somehow related, such as propensity to nicotine addiction and lung cancer. In either case, their impact on organismal fitness may or may not be correlated (the same allele can have big or small, positive or negative, effects on a visible phenotype and disease susceptibility), which makes modeling of the effect of evolutionary processes on allele frequencies even harder.

Nevertheless, a convenient framework for accommodating pleiotropy and purifying selection, first introduced by R.A. Fisher almost a century ago, is the geometric model [19]. If we imagine all SNPs that reduce fitness by some value *s* as lying equidistant in two-dimensional fitness space from the optimum, they would describe a circle around the optimal center with radius proportional to *s*. Drawing a new effect on a second trait from any point on the circle, there is a slightly greater chance that the arrow points outside than inside the circle; in other words, it is more likely to decrease fitness than increase it, and similarly, with respect to a sphere in three dimensions, and so forth. Pleiotropy tends to reduce the advantage of even beneficial variants at a focal trait, and this “multivariate” nature of genetics is partly what ensures that only small effect alleles become common. Much mathematical work has explored this model (e.g., [20–22]), including influential work on “adaptive walks,” which it turns out can be dominated by relatively large-effect alleles [23]. Until now, though, theory relating the model to stable infinitesimal traits has been lacking.

A corollary of the geometric model is that under pleiotropy, the distribution of allelic effects influencing a trait of interest can be very different from that expected if selection acted only on that trait. Previous research has explored these distributions under various assumptions, but the new paper derives mathematical expectations from first principles and then checks the conclusions against the most recent GWAS conclusions for height [24] and BMI [25]. Two key results are that the majority of discovered variants in GWAS are likely to be experiencing relatively strong purifying selection and that the expected variance explained by these sites is a simple function of the effect of the allele on fitness divided by the combination of the degree of pleiotropy and the effective population size. Because allele frequencies covary with the selection differential (Fig 1B), strongly selected variants are predicted to have similar effects on the trait, the magnitude of which is set by the variance for fitness and the mutational target size. This model provides a better fit to the empirical data than does the assumption of direct selection on the trait, and the 2–3-fold difference in heritability (as well as number of variants discovered) between height and BMI is mostly attributed to differences in the greater number of loci in which mutations will affect height and fitness than BMI and fitness.

It should be emphasized that strong selection is a relative term: scaled by the effective population size, selection differentials need only be greater than about 10^−3^ in a population of 10,000 individuals, which is thought to represent most of human history. Such a differential is much smaller, for example, than de novo mutations that are causal in schizophrenia [26], and much smaller than effects field zoologists could expect to measure as organisms adapt to a new niche [27]. These results serve to remind us that the bulk of the genetic variation segregating in natural populations is due to alleles of very small effect, rather than the types of large-effect sites that tend to be promoted as representative examples of adaptive loci.

Simons et al [2] should be regarded as a framework for further theoretical development and hypothesis testing. They provide one example at the end of the paper, with which they argue that much of the variation affecting disease risk in Europeans will derive from alleles that arose in the bottleneck before *Homo sapiens* spread across and out of Africa, which in turn predicts an age distribution for GWAS hits that can be tested [28]. Humans are somewhat unusual as species go in terms of our ability to occupy diverse ecological niches and, most recently, to construct our own environment, for better or for worse. How many of the traits we measure and are interested in relate to historical fitness is difficult to know, which might in turn sometimes affect the expectations derived from this theory. More generally, though, we now have a good explanation for why tens of thousands of variants influence quantitative traits, and a way forward to explain differences in genetic architecture among traits, among populations, and even among species.

## Abbreviations

GWAS: genome-wide association study
QTL: quantitative trait loci
sdu: standard deviation unit
